# Spectrum of musculo-skeletal disorders in sickle cell disease in Lagos, Nigeria

**DOI:** 10.1186/1749-799X-5-2

**Published:** 2010-01-18

**Authors:** Rufai A Balogun, Dike C Obalum, Suleiman O Giwa, Thomas O Adekoya-Cole, Chidiebere N Ogo, George O Enweluzo

**Affiliations:** 1 Department of Surgery, College of Medicine, University of Lagos (CMUL)/Lagos University Teaching Hospital (LUTH), PMB 12003, Lagos, Nigeria

## Abstract

**Background:**

Sickle cell anemia (SCA) is a common genetic disease in Nigeria. Past studies from West Africa focused on isolated aspects of its medical and surgical presentations. To the best of our knowledge, the musculo-skeletal presentations amongst Nigerians with SCA have not been documented in a single all encompassing study. This work aims to prospectively document the musculo-skeletal disease burden among SCA patients.

**Methods:**

In a prospective study of 318 consecutive patients with genotype-confirmed SCA at the Lagos University Teaching Hospital (LUTH), the musculo-skeletal pathologies, anatomic sites, grade of disease, age at presentation and management outcome were recorded over a one-year period. Data obtained were analyzed using Epi-Info software version 6.0. Data are presented as frequencies (%) and mean values (SD) as appropriate.

**Results:**

The HbSS genotype occurred in 296 (93.0%), while 22 (7.0%) were HbSC. 100 (31.4%) patients with average presenting haemoglobin concentration of 8.2 g/100 ml in the study group, presented with 131 musculo-skeletal pathologies in 118 anatomic sites. Osteomyelitis 31 (31%) and septic arthritis 19 (19%) were most commonly observed in children less than 10 years. Skin ulcers and avascular necrosis (AVN) occurred predominantly in the older age groups, with frequencies of 13 (13.0%) and 26 (26.0%) respectively. 20 (71.5%) of diagnosed cases of AVN presented with radiological grade 4 disease. The lower limbs were involved in 84 (71.1%) of sites affected. Lesions involving the spine were rare 11 (0.9%). Multiple presentations occurred in 89 (28.0%) of patients; 62 (69.7%) of which were children below 10 years.

**Conclusions:**

Musculo-skeletal complications are common features of sickle cell anaemia seen in 31.4%. Infectious aetiologies predominate with long bones and joints of lower limbs more commonly affected by osteomyelitis and septic arthritis. Healthcare providers managing SCA should be aware of the potential morbidity and mortality of these conditions to ensure early diagnosis and adequate management.

## Background

Sickle cell disease (SCD) is a group of inherited haemoglobinopathies occurring mainly in Negroid populations in and out of Africa, characterized by a predominance of haemoglobin S (HbS) in the erythrocytes [1]. It was first recognized by James B. Herrick [2] in 1910 when he described abnormal sickle-shaped cells in an anaemic patient of Negroid extraction. Pauling et al [3] discovered the presence of abnormal haemoglobin in patients with sickle cell disease in 1949. SCD is the most frequent haemoglobinopathy in the world [4,5] and currently the second most common genetic disease after Down's syndrome [5]. Sickle cell disease is said to affect between 2-3% of the Nigerian population [1].

Sickle cell anaemia (SCA) occurs when there is homozygote HbSS or composite heterozygote HbSC [1]. It is primarily a disease of haemopoetic system in which the skeleton bears the brunt of its complications [6]. Bone changes in SCA occur due to marrow hyperplasia, tissue ischaemia and infarction due to vaso-occlusion [7-9]. Musculo-skeletal manifestations constitute up to 80% of indications for presentation in hospital in SCA during their life time [10-14]. Pain is the principal complaint either acute following skeletal or soft tissue infarction or chronic secondary to avascular necrosis of bone at various joints [15]. Most studies of musculo-skeletal presentations of SCA in Nigeria have focused on selected disease conditions [8,10,16-19].

SCA causes a heavy burden on the society by the high morbidity and premature death associated with it [20]. This study was designed to prospectively document, using a comprehensive approach, the spectrum and frequency of musculo-skeletal presentations among patients with SCA. This would provide useful data on the burden of musculo-skeletal disease in SCA, for further research, infrastructural and manpower planning towards appropriate care delivery.

## Methods

This prospective study was conducted over a 12-month period between June 2000 and May 2001 at Lagos University Teaching Hospital (LUTH), Lagos, Nigeria. LUTH is one of the foremost tertiary hospitals in Nigeria. The study protocol was approved by the Health Research and Ethics Committee of the hospital. Informed consent was obtained from all study participants or their proxies.

Cases included were consecutively presenting patients with Hb genotype SS or SC attending one of 4 sites in the hospital: orthopaedic outpatient clinic, accident and emergency department, haemotology clinic (adult SCD clinic) and paediatric outpatient department. Through a pre-arranged notification network, the investigators were informed of any case of SCA presenting during the study period, and each patient was then evaluated by one of the investigators using the standardized format developed for the study. Musculo-skeletal complaints were defined as any problem affecting bones or/and its associated soft tissues. Patients with SCA with congenital musculo-skeletal anomalies were excluded.

Clinical evaluation and relevant investigations were carried out as part of standard management of these patients to arrive at a diagnosis by the authors. The diagnosis of AVN was made using plain x-rays as the institution at the time of study had no facilities for CT, MRI or Isotope scan. This was stated as the main reason for the absence of stage 1 disease in our study. All patients with a diagnosis of septic arthritis had emergency arthrotomy and drainage. Aspirates were sent for culture and antibiotic sensitivity. Active ulcers were considered as ulcers but those with unspecific scars on the ankles were not considered as such. Osteomyelitis was diagnosed based on clinical evaluation, needle puncture and intra operative aspirate of purulent fluid with positive cultures.

A standard proforma was filled following detailed history and physical examination with requisite investigations to confirm the diagnosis. Variables recorded included age, sex, weight, height, genotype, anatomic site(s) involved, clinical features and the diagnosis.

Data obtained were analyzed using Epi-Info software version 6.0. Data are presented as frequencies (%) and mean values (SD) as appropriate, and compared using either the chi square test (for proportions) or student's T test for mean values. P < 0.05 is taken as statistically significant.

## Results

### Demography

Three hundred and eighteen patients with SCA were studied. 100 (31.4%) patients with average presenting haemoglobin concentration of 8.2 g/100 ml had 131 musculo-skeletal presentations at 118 anatomic sites. 60 (60.0%) of these were males and 40 (40.0%) were females, giving a male: female ratio of 1.5:1. The ages of all the patients ranged between 1 and 45 years, with a mean of 14.2 ± 11.5 years. Forty-six (46.0%) of the patients were aged below 10 years, while 52 (52.0%) were aged between 11 - 40 years. Two (2.0%) were aged above 40 years. Of the 100 patients with musculo-skeletal features studied, 93(93.0%) had HbSS genotype while 7(7.0%) had SC. (Table 1).

**Table 1:** Demographic parameters and genotype of patients with musculo-skeletal presentation

Parameter	No	%
Males,	60	60.0%
Female,	40	40.0%
M: Female ratio	1.5:1	-
		
Age range, yrs	1 - 45	-
Mean age, yrs	14.2 ± 11.5	-
		
Age (yrs)		
< 10	46	46.0%
11 - 20	27	27.0%
21 - 30	18	18.0%
31 - 40	7	7.0%
> 40	2	2.0%
		
Genotype		
SS	93	93.0%
SC	7	7.0%

### Infectious and non-infectious spectrum of musculoskeletal disorders

**Figure 1:**
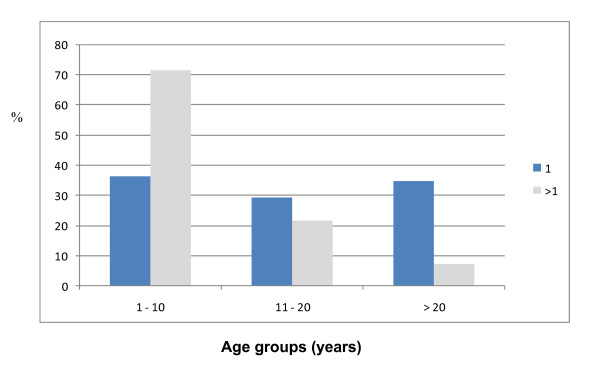
**Frequency (%) of multiple and single musculoskeletal presentations by age group of patients in SCA**. Multiple presentations (>1) predominate in age group 1-10 years.

Table 2 shows the frequency distribution of 131 musculo-skeletal presentations of SCD documented. Osteomyelitis accounted for 49 (37.4%), followed by avascular necrosis (AVN) 28 (21.4%) and septic arthritis 20 (15.3%). 25 (51.0%) of osteomyelitis were caused by staphylococcus species, 16 (32.7%) by salmonella, 4 (8.2%) by haemophilus and 3 (6.1%) by streptococcus. No organism was isolated in one case. All cases of AVN affected the femoral head presenting in Ficat's grade 4 disease (additional file 1: Figure S2) in 20 (71.5%) cases, 7 (25.0%) in grade 3, 1 (3.5%) in grade 2 (additional file 1: Figure S2), while no patient presented with grade 1 disease. Table 3 shows the age distribution of the various presentations. Osteomyelitis (p = 0.00002), septic arthritis (p = 0.000005) and pathological fractures (p = 0.049) were significantly more common in patients under the age of 10 years, while AVN (p = 0.000007) and leg ulcers (p = 0.00001) were significantly more common in older ages. Multiple presentations of SCA were observed in 28 (28.0%) patients. As shown in Figure 1, 20 (71.4%) of these multiple presentations occurred in patients aged less than 10 years, 6 (21.4%) in those aged between 11 and 20 years and 2 (7.2%) in those over 20 years (p = 0.003).

**Table 2:** Distribution of musculo-skeletal presentations of patients

Disorder	Frequency	Percentage
Dactylitis	10	7.6
Osteomyelitis	49	37.4
Septic Arthritis	20	15.3
Ulcers	14	10.7
Avascular necrosis	28	21.4
Pathological fracture	10	7.6
**Total**	**131**	**100**

**Table 3:** Distribution by age of presentations

Disorder	<10 years N (%)	11-20 N (%)	> 20 N (%)	Total	P-value
Dactylitis	10	-	-	10	N/A
Osteomyelitis	31 (63.3)	15 (30.6)	3 (6.1)	49	P = 0.00002
Septic Arthritis	19 (95.0)	1 (5.0)	-	20	P = 0.000005
Ulcers	1 (7.1)	2 (41.3)	11 (78.6)	14	P = 0.00001
Avascular necrosis	2 (7.2)	13 (46.4)	13 (46.4)	28	P = 0.000007
Pathological	8 (80.0)	2 (20.0)	-	10	P = 0.049
**Total**	**71**	**33**	**27**	**131**	

### Regional anatomic location of musculo-skeletal presentations

One hundred and eighteen sites were involved in the study population with 84 (71.2%) occurring in the lower limbs, 33 (28.0%) in upper limbs and 1 (0.8%) in the spine. An analysis of the pattern of regional anatomic involvement according to age is shown in Table 4. 25 (75.8%) of the cases in the upper limb occurred in patients aged less than 10 years, with 8 (24.2%) occurring in other age groups. These differences were found to be statistically significant with a p-value of 0.00008. Osteomyelitis affected 49 cases, with the distribution as follows: femur 20 (40.8%), tibia 14 (28.6%), humerus 11 (22.0%) and radius 4 (8.2%). Septic arthritis was found in the hip joint in 8 (40.0%) cases, followed by the knee 5 (25.0%), elbow 4 (20.0%), shoulder 2 (10.0%) and ankle 1(0.5%). The humerus was pathologically fractured in 5 (50.0%), femur (2/10), tibia (2/10) and radius (1/10) were similarly affected.

**Table 4:** Distribution by age of regional anatomic involvement

Site	<10 years N (%)	11 - 20 years N (%)	11 - 20 years N (%)	Total (%)
Upper Limbs	25 (75.8)	6 (18.2)	2 (6.0)	33 (28.0)
Lower Limbs	33 (39.2)	26 (31.0)	25 (29.8)	84 (71.1)
Spine	-	-	1 (0.09)	1 (0.09)
**Total**	**58 (49.2)**	**32 (27.1)**	**28 (23.7)**	**118 (100)**

## Discussion

The natural history of SCA is associated with a high morbidity and mortality [20], although close surveillance, prevention, and early detection of complications can improve outcomes. High morbidity and mortality in SCA is due, in part, to the increased proneness to infection [1], particularly in our environment where communicable diseases are prevalent. In this present study, it is thus not surprising that the most frequently encountered underlying aetiology of musculoskeletal presentations was infection. Presentations directly related to infections range between 11 - 61% in various studies [8,12,21]. The increased predisposition to infection has been attributed to several factors, prominent among which are defective immune mechanism and functional asplenia [1,16,18,22]. Meticulous care for these patients as well as improved health promotion and health seeking behavior would reduce the morbidity and mortality of this primeval condition.

Of 318 patients with SCA we studied, 31.4% had musculo-skeletal presentations. This figure is lower than that reported by Benneth and Namyak in 1990[12]. The male preponderance found in this study is in keeping with previous studies [12-14]. An overwhelming majority of patients in this study were below 40 years with only 2% over that age. This may be due to reduced life expectancy in SCA patients as had been documented in Cameroun [23] and Senegal [24]. This differs sharply from findings in the United States where 50% were over 40 years [25]. Poor life expectancy among SCA patients in sub-saharan Africa may be related to factors like the absence of hydroxyurea therapy that may improve survival [26] or low educational attainment, poverty and limited access to medical facilities among these patients [27]. The predominance of young patients may also be due to differences in health seeking behaviour between the younger, more active persons with SCA, and the older patients with SCA. The higher frequency of HbSS genotype in this study is in keeping with earlier reports which showed that this is the commonest variant of SCA among Nigerians [13]. Our observed 7.0% frequency of HbSC had been previously reported in West Africa [13].

Osteomyelitis is a major presentation of SCA and accounted for one-third of cases in this study. This is however lower than 61.0% reported among Saudis [12] but comparable to 29.0% reported by Mijiyawa in a neigbouring West African country [28]. The femur and tibia were the most frequently involved bones followed by the humerus. This pattern had been reflected in other studies [16,29,30]. Septic arthritis, another major infective presentation is reported to represent 6-11% of bone and joint manifestations of SCA [11,31,32]. The relatively higher value of 15.3% found in this study may not be unrelated to the preponderance of young patients, who are more prone to infections; low socioeconomic status [33,34] and the poor sanitary living conditions of most of these patients [33], as well as ineffective enforcement of environmental sanitation laws in our environment.

SCA is the commonest cause of AVN in Nigeria [9,17]. AVN complicating SCA has previously been reported to occur in 3 - 19% of SCA patients [12,17]. In this study, a higher percentage was recorded, mostly presenting in late stages as was the case in a Yaounde study by Bahebeck et al [23] in 2004. This may be because first line medical care givers missed the diagnosis at earlier stages. It may also be due to patients presenting first to traditional bone setters, churches and mosques only to come to hospitals at late stages. Lack of modern diagnostic facilities and/or relatively high cost of orthodox medical care in Nigeria for most of these patients may have contributed to this. There is therefore a need to mount education and awareness campaigns for the sickle cell disease population, their medical care givers and the society in general on the need to seek and give appropriate care early. This is because there is no doubt that there is upward surge in life expectancy of SCA patients due to better understanding and correct management of the complications [6]. Also provision of modern diagnostic facilities such as magnetic resonance imaging at affordable costs and with improved accessibility would help in the recognition of the early stages of this disease.

Pathological fractures were seen in 7.6% of our patients, a figure higher than 4% reported by Omojola et al [17]. Surprisingly, there was a preponderance of affectation of the humerus compared to the femur and tibia, despite the fact that most presentations were seen in the lower limbs. The smaller number of fractures in the lower limbs may be ascribed to the compulsive reduction in physical activity of the lower limbs during periods of significant bone pain, while patients may continue the use of upper limbs even with severe disease and pain.

We found a statistically significant relationship between infectious presentations such as osteomyelitis and septic arthritis with less than 10 year olds, as well as between non-infectious presentations such as AVN and skin ulcers with older patients. AVN and skin ulcers are mostly due to progressive devascularisation of affected areas. Their preponderance in older patients may result over the years of life from chronic anaemia causing marrow hyperplasia as well as red cell sickling secondary to hypoxia leading to bone infarcts [35]. These infarcts are typically in areas supplied by end arteries [35].

## Conclusions

This study has shown that osteomyelitis remains the most common musculo-skeletal presentation of SCA and occurs predominantly in patients below the age of 10 years. The predominant presentation in adolescents is AVN, with majority of them presenting in the late stage. Multiple presentations are seen in all groups and this calls for a detailed assessment of SCA patients by health care professionals in order to avoid cases of missed diagnosis.

## Competing interests

The authors declare that they have no competing interests.

## Authors' contributions

RAB contributed to conception, design, acquisition, analysis and interpretation of data. DCO is the corresponding author, he contributed to conception, design, acquisition, analysis and interpretation of data as well as intellectual content and manuscript writing. SOG contributed to interpretation of data, intellectual content and manuscript writing. TOA contributed to conception, design, interpretation of data and intellectual content. All authors read and approved the final manuscript. CNO and GOE contributed to data acquisition.

## Authors' information

RAB: MBBS, FMCS. Lecturer/Consultant

SOG: MBBS, FMCS, FWACS, FICS. Senior lecturer/Consultant

DCO: MBBS, FMCS, FWACS, FICS. Senior lecturer/Consultant

TOA: MBBS, FRCS, FWACS. Lecurer/Consultant

CNO: MBBS, FWACS, Consultant

GOE: MBBS, FWACS, FMCS, Consultant

## Supplementary Material

Additional file 1 **Additional radiograph figures**. Figure S1 - Antero-posterior plain radiograph of the pelvis showing stage III. AVN on the right hip and stage II AVN on the left hip. Figure S2 - Antero-posterior plain radiograph of the pelvis showing stage IV. AVN on the right hip. Click here for file
