# Supplementary material for: Spectrum of musculo-skeletal disorders in sickle cell disease in Lagos, Nigeria
Source: J Orthop Surg Res. 2010 Jan 18;5:2. doi: 10.1186/1749-799X-5-2 (PMC2821293; doi:10.1186/1749-799X-5-2)

Additional files

Additional file 1

Title : Antero-posterior plain radiograph of the pelvis showing stage III

AVN on the right hip and stage II AVN on the left hip.


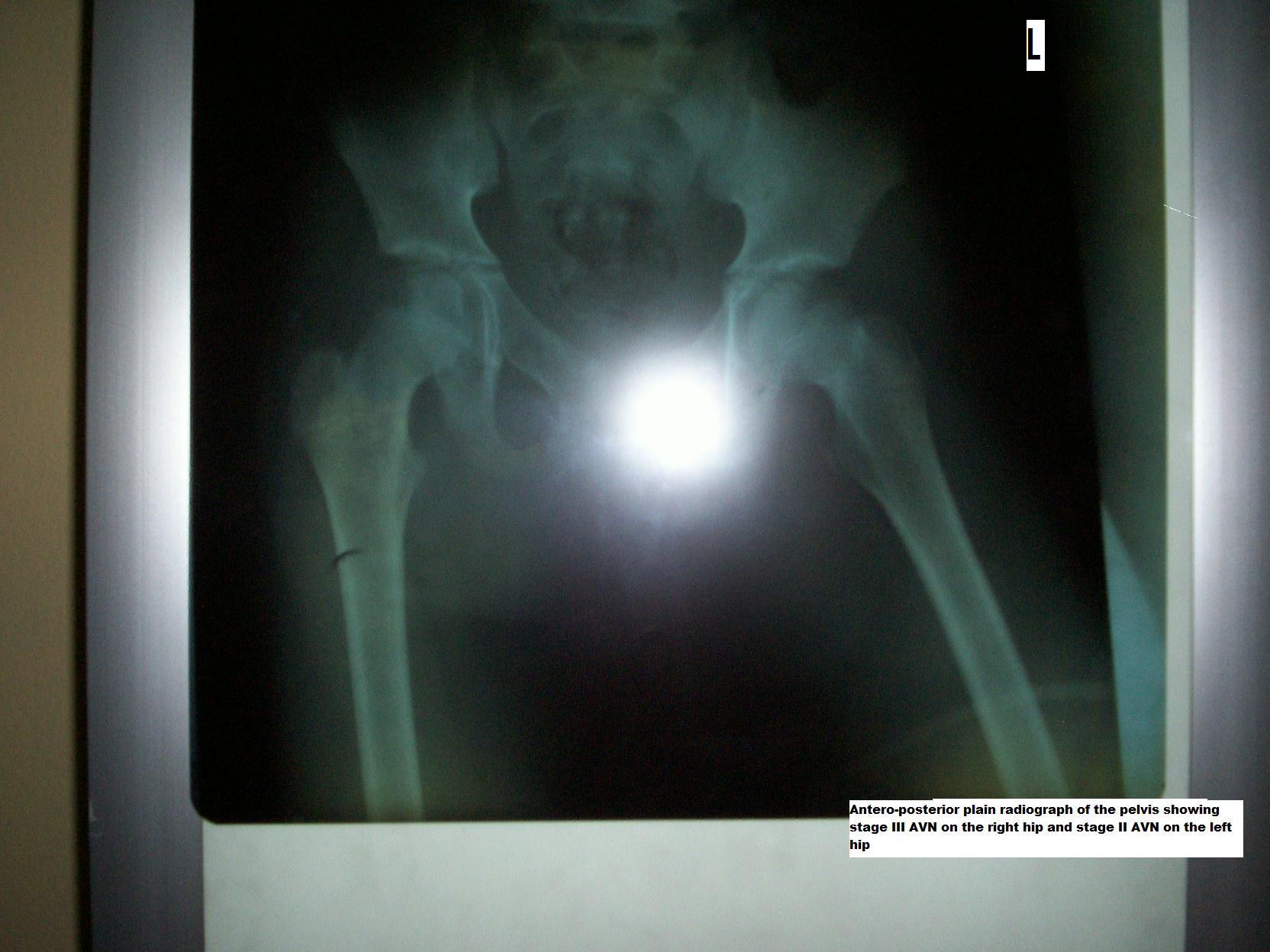


Additional file 2

Title : Antero-posterior plain radiograph of the pelvis showing stage IV

AVN on the right hip.


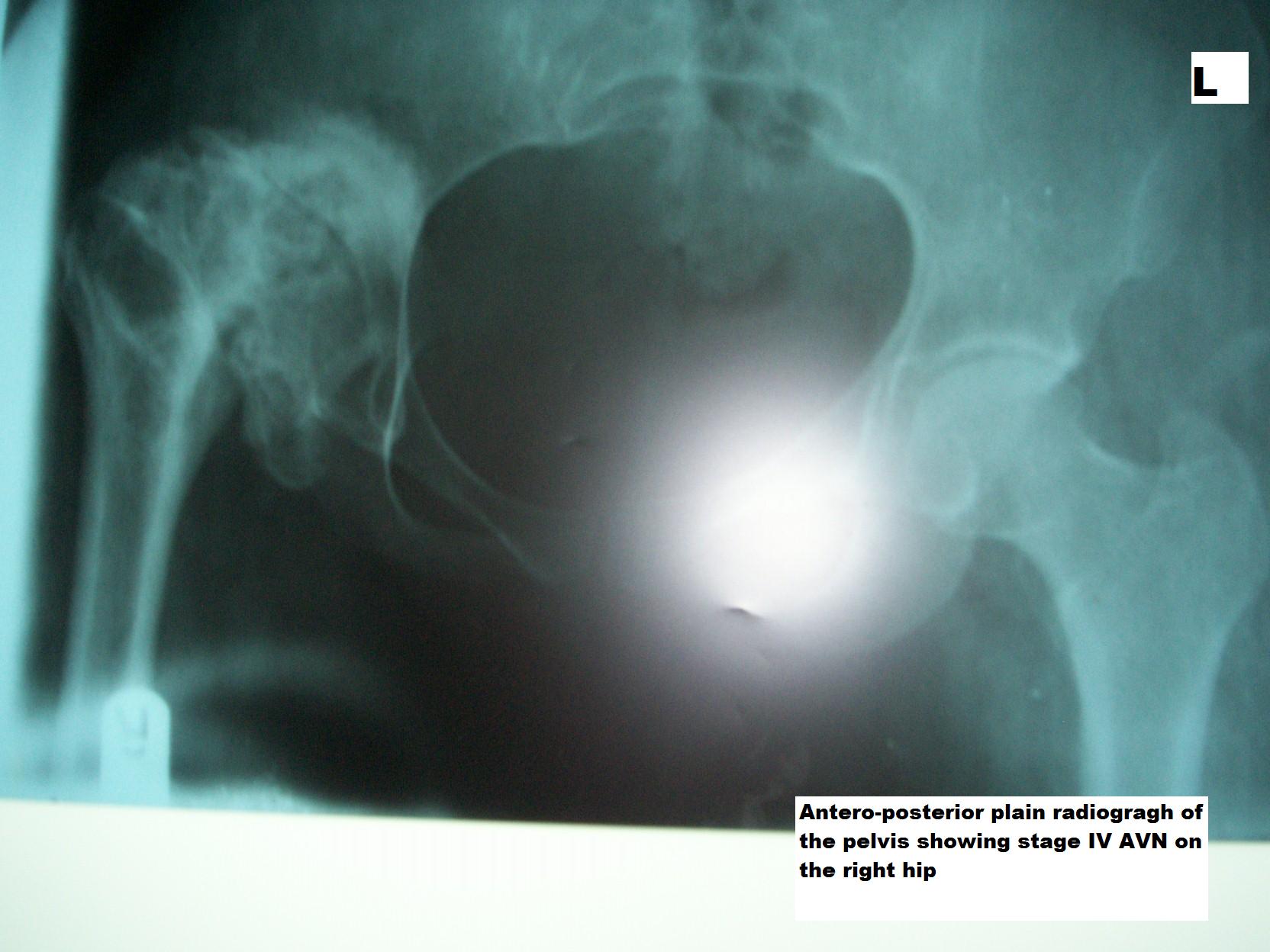

Supplement: Additional file 1 — Additional radiograph figures. Figure S1 - Antero-posterior plain radiograph of the pelvis showing stage III. AVN on the right hip and stage II AVN on the left hip. Figure S2 - Antero-posterior plain radiograph of the pelvis showing stage IV. AVN on the right hip. [file 1749-799X-5-2-S1.DOC]
